# Supplementary material for: Modeling hereditary diffuse leukoencephalopathy with axonal spheroids using microglia-sufficient brain organoids
Source: eLife. 2025 May 21;13:RP96693. doi: 10.7554/eLife.96693 (PMC12094700; doi:10.7554/eLife.96693)
Supplement: Supplementary file 1. [file elife-96693-supp1.docx]

**Table 1: Demographics of HDLS patients**

| **HD #** | **Sex** | **Age** | **Mutation** |
| --- | --- | --- | --- |
| 1 | M | 42 | Exon 18; c.2381T>C（Ile794Thr） |
| 2 | F | 32 | Exon 15; c.2026C>T (p.Arg676Ter) |

**Table 2:** **Primers used for cytokine expression**

| **Name** | **Forward Primer** | **Reverse Primer** |
| --- | --- | --- |
| Human IFN-g | TCGGTAACTGACTTGAATGTCCA | TCGCTTCCCTGTTTTAGCTGC |
| Human TNF-α | CCTCTCTCTAATCAGCCCTCTG | GAGGACCTGGGAGTAGATGAG |
| Human IL-1β | ATGATGGCTTATTACAGTGGCAA | GTCGGAGATTCGTAGCTGGA |
| Human IL-18 | TCTTCATTGACCAAGGAAATCGG | TCCGGGGTGCATTATCTCTAC |
| Human IL-10 | GACTTTAAGGGTTACCTGGGTTG | TCACATGCGCCTTGATGTCTG |
| Human TGF-β | GGCCAGATCCTGTCCAAGC | GTGGGTTTCCACCATTAGCAC |
| Human IL-6 | ACTCACCTCTTCAGAACGAATTG | CCATCTTTGGAAGGTTCAGGTTG |
| Human IL-12b | ACCCTGACCATCCAAGTCAAA | TTGGCCTCGCATCTTAGAAAG |
| HPRT1 | TGACCAGTCAACAGGGGAC | TGCCTGACCAAGGAAAGC |
|  |  |  |
| Mouse IFN-g | GCCACGGCACAGTCATTGA | TGCTGATGGCCTGATTGTCTT |
| Mouse TNF-α | CAGGCGGTGCCTATGTCTC | CGATCACCCCGAAGTTCAGTAG |
| Mouse IL-1β | GAAATGCCACCTTTTGACAGTG | TGGATGCTCTCATCAGGACAG |
| Mouse IL-18 | GTGAACCCCAGACCAGACTG | CCTGGAACACGTTTCTGAAAGA |
| Mouse IL-10 | CTTACTGACTGGCATGAGGATCA | GCAGCTCTAGGAGCATGTGG |
| Mouse TGF-β | CCACCTGCAAGACCATCGAC | CTGGCGAGCCTTAGTTTGGAC |
| Mouse IL-6 | CTGCAAGAGACTTCCATCCAG | AGTGGTATAGACAGGTCTGTTGG |
| Mouse IL-12b | GTCCTCAGAAGCTAACCATCTCC | CCAGAGCCTATGACTCCATGTC |
| GAPDH | AGGTCGGTGTGAACGGATTTG | GGGGTCGTTGATGGCAACA |

Expression of target genes were normalized to GAPDH quantified in parallel amplification reactions

**Table 3: List of Reagents**

| **Reagent** | **Source** | **Identifier** |
| --- | --- | --- |
| Dulbecco’s Modified Eagle Medium (DMEM) | Corning | Cat # 10-014-CV |
| Fetal Bovine Serum | Gibco | Cat # 26140079 |
| Penicillin/Streptomycin | Gibco | Cat # 15140122 |
| Antibiotic-Antimycotic | Gibco | Cat # 15240062 |
| Phosphate buffered saline | Corning | Cat # 21-040-CV |
| TeSR-E7 | Stemcell | Cat # 5914 |
| Epi5 Episomal reprogramming kit | Invitrogen | Cat # A15960 |
| mTeSR-1 | Stemcell | Cat # 85850 |
| Matrigel | Corning | Cat # 354234 |
| TrueCut Cas9 Protein V2 | Thermofisher | Cat # A36498 |
| CloneR | Stemcell | Cat # 05888 |
| Rock inhibitor; Y-27632 (Dihydrochloride) | Stemcell | Cat # 72302 |
| Human stem cell nucleofector starter kit | Lonza | Cat # VPH-5002 |
| Human dermal fibroblast nucleofector kit | Lonza | Cat # VPD-1001 |
| PSC 4-Marker Immunocytochemistry Kit | Thermofisher | Cat # A24881 |
| StemPro-34 SFM | Gibco | Cat # 10639011 |
| IMDM (Iscove's Modified Dulbecco's Medium) | Gibco | Cat # 1980030 |
| Ham's F-12 Nutrient Mix | Gibco | Cat # 11765054 |
| DMEM/F-12 | Gibco | Cat # 11320033 |
| L-glutamine | Gibco | Cat # 25030081 |
| Ascorbic acid | Sigma | Cat # A4544 |
| MTG (1-Thioglycerol) | Sigma | Cat # M6145 |
| Human Transferrin | Roche | Cat # 10-652-202-001 |
| CHIR99021 | Sigma | Cat # SML1046-5MG |
| Recombinant Human BMP-4 | Peprotech | Cat # 120-05 |
| Recombinant Human VEGF | Peprotech | Cat # 100-20 |
| Recombinant Human FGF-basic | Peprotech | Cat # 100-18B |
| Recombinant Human DKK-1 | Peprotech | Cat # 120-30 |
| Recombinant Human SCF (c-kit) | Peprotech | Cat # 300-07 |
| Recombinant Human IL-3 | R&D Systems | Cat # 203-IL |
| Recombinant Human IL-6 | R&D Systems | Cat # 206-IL |
| Recombinant Human M-CSF | Peprotech | Cat # 300-25 |
| N-2 Supplement (100X) | Gibco | Cat # 17502048 |
| B27 Supplement (50X) | Gibco | Cat # 17504044 |
| Bovine serum albumin | Yeasen | Cat # 36101ES76 |
| KnockOut Serum Replacement | Gibco | Cat # 10828028 |
| GlutaMAX Supplement | Gibco | Cat # 35050061 |
| MEM Non-Essential Amino Acids Solution (100X) | Gibco | Cat # 11140050 |
| 2-Mercaptoethanol | Gibco | Cat # 21985023 |
| Dorsomorphine | Sigma | Cat # P5499 |
| A-83 | Sigma | Cat # SML0788-5MG |
| SB-431542 | Sigma | Cat # S4317-5MG |
| Human insulin | Absin | Cat # abs42225219 |
| Triton X 100 | MERCK | Cat # X100 |
| RapiClear | SUNJin Lab | Cat # RC147001 |
| SMART-Seq HT kit | Takara | Cat # 634437 |
| TRIzol Reagent | Invitrogen | Cat # 15596018 |
| VAHTS DNA Clean beads | Vazyme | Cat # N411-01-AA |
| Qubit dsDNA HS Assay Kit | Invitrogen | Cat # Q33231 |
| TruePrep DNA library prep kit V2 for illumina | Vazyme | Cat # TD502-02 |
| MTT test | Absin | Cat # abs50010-500T |
| Wright-Giemsa staining kit | BASO | Cat # BA-4017 |
| pHrodo Red *E.coli* bioparticles conjugate | Invitrogen | Cat # P35361 |
| PrimeScript RT Reagent Perfect Real Time kit | Takara | Cat # RR037B |
| ChamQ SYBR® Color qPCR Master Mix Low ROX Premixed | Vazyme | Cat # Q431-02 |
| Paraformaldehyde | Yuanye Bio | Cat # r20497 |
| TrypLE Express | Gibco | Cat # 12604013 |
| ReLeSR | Stemcell | Cat # 100-0483 |
| Accutase | Stemcell | Cat # 07920 |
| GlycoBlue Coprecipitant | Thermofisher | Cat # AM9515 |
| BD IMag™ Anti-CD11b Magnetic Particles - DM | BD Biosciences | Cat # 558013 |
| Percoll | Cytiva | Cat # 17089101 |
| IC Fixation Buffer | Invitrogen | Cat # 00-8222-49 |
| Permeabilization Buffer 10X | Invitrogen | Cat # 00-8333-56 |
| DAPI | ThermoFisher | Cat # D1306 |
| MycoStrip | InvivoGen | Cat # rep-mys-10 |

**Table 4: Cell culture medium**

| **Medium** | **Composition** |
| --- | --- |
| Human dermal fibroblast medium | DMEM supplemented with 10% FBS, 1% Antimycotics/Antibiotics |
| StemPro-34 SFM Medium | Stempro-34 SFM, supplemented with 200 ug/mL Human Transferrin, 2 mM Glutamic Acid, 1x Pen/Strep (P/S), 0.5 mM Ascorbic Acid and 0.45 mM MTG. |
| Serum-Free Differentiation Medium (SF-Diff) | 75% IMDM with Glutamax, 25% F12, 1x N2 supplement, 1x B27 Supplement, 0.05% BSA and 1x Pen/Strep. |
| Forebrain organoid first medium (F1M) | DMEM/F12 supplemented with 20% Knockout serum, 1x GlutaMax, 1x MEM-NEAA, 1x 2-Mercaptoethanol, 1x Pen/strep, Dorsomorphine 2 uM, A-83 2 uM |
| Forebrain organoid second medium (F2M) | DMEM/F12 supplemented with 1x N2 supplement, 1x GlutaMax, 1x MEM-NEAA, 1x Pen/Strep, CHIR-99021 1 uM, SB-431542 1 uM |
| Forebrain organoid third medium (F3M) | DMEM/F12 supplemented with 1x N2 supplement, 1x B27 supplement, 1x GlutaMax, 1x MEM-NEAA, 1x 2-Mercaptoethanol, 1x Pen/Strep, Insulin 2.5 ug/ml |

**Table 5: Antibody list**

| **Antibody** | **Source** | **Identifier** | |
| --- | --- | --- | --- |
| Rabbit anti-SOX2 | Abcam | Cat # Ab97959 | |
| Goat anti IBA-1 | Abcam | Cat # Ab5076 | |
| Mouse anti-NESTIN | Merck | Cat # MAB5326 | |
| Mouse anti-CD15 (SSEA-1) | Biolegend | Cat # 323033 | |
| Mouse anti-CD24 | BD Biosciences | Cat # 561646 | |
| Mouse anti-CD44 | Biolegend | Cat # 338805 | |
| Mouse anti-CXCR4 (CD184) | Biolegend | Cat # 306515 | |
| Mouse anti-CD271 | BD Biosciences | Cat # 743358 | |
| Alexa-Fluor ® 488-conjugated Goat anti-mouse | Abcam | Cat # Ab150135 | |
| Alexa-Fluor ® 594-conjugated Goat anti-rabbit | Abcam | Cat # Ab150080 | |
| Alexa-Fluor ® 647-conjugated Donkey anti-goat CFSE | Abcam | Cat # Ab150135 | |
| Annexin V - PE | BD Pharmingen | Cat # 559763 | |
| Rabbit anti-OCT4 | Invitrogen | Cat # A24867 | |
| Mouse anti-SSEA4 | Invitrogen | Cat # A24866 | |
| Rat anti-SOX2 | Invitrogen | Cat # A24759 | |
| Mouse anti-TRA-1-60 | Invitrogen | Cat # A24868 | |
| Alexa Fluor™ 594 donkey anti-rabbit | Invitrogen | Cat # A24870 | |
| Alexa Fluor™ 488 goat anti-mouse IgG3 | Invitrogen | Cat # A24877 | |
| Alexa Fluor™ 488 donkey anti-rat | Invitrogen | Cat # A24876 | |
| Alexa Fluor™ 594 goat anti-mouse IgM | Invitrogen | Cat # A24872 | |
| Anti-mouse CD115 BV605 (AFS98) | Biolegend | Cat # 135517 |  |
| Anti-mouse Ly6G BV711 (1A8) | Biolegend | Cat # 127643 |  |
| Anti-mouse Siglec F Alexa Fluor 647 (E50-2440) | BD Biosciences | Cat # 562680 |  |
| Anti-mouse CD11b BV650 | Biolegend | Cat # 139309 |  |
| Anti-mouse CD172a PerCP-eFluor® 710 (P84) | Biolegend | Cat # 46-1721-82 |  |
| Anti-mouse Ly6C BV785 (HK1.4) | Biolegend | Cat # 128041 |  |
| Anti-mouse CD45 BUV395 (30-F11) | BD Biosciences | Cat # 565967 |  |

|  | **sgRNA** | **ssDNA** |
| --- | --- | --- |
| **HD1** | 5’˗TGGTCATGTGGCCAAGACTG˗3’ | 5’˗GCGCGTAACGTGCTGTTGACCAATGGTCATGTAGCTAAAATCGGGGACTTCGGGCTGGCTAGGGACATCATGAATGACTC˗3' |
| **HD2** | 5'˗GCTCAACTTTCTGTGAAGGA˗3' | 5’˗CACGGAGTACTGTTGCTATGGCGACCTGCTCAATTTCCTTCGAAGAAAAGCTGAAGCCATGCTGGGACCCAGCCTGAGCC˗3' |

**Table 6: sgRNA and ssDNA template used for targeting HD1 and HD2**

**(**Green nucleotide denotes a silent mutation)
